# Monocyte-regulated interleukin 12 production drives clearance of *Staphylococcus aureus*

**DOI:** 10.1371/journal.ppat.1012648

**Published:** 2024-10-17

**Authors:** Adeline Peignier, Jisun Kim, Alexander Lemenze, Dane Parker

**Affiliations:** Department of Pathology, Immunology and Laboratory Medicine, Center for Immunity and Inflammation, Rutgers New Jersey Medical School, Newark New Jersey United States of America; University of Illinois at Chicago College of Medicine, UNITED STATES OF AMERICA

## Abstract

*Staphylococcus aureus* is a versatile bacterium responsible for conditions ranging from mild skin and soft-tissue infections to serious disorders such as pneumonia and sepsis. Monocytes play a role in protection against pathogens by migrating to inflamed tissues and differentiating into macrophages but their specific role in the context of *S*. *aureus* pulmonary infection has not been fully elucidated. Using a CCR2-DTR transgenic mouse model we demonstrate that over the course of infection monocyte depletion resulted in worse airway clearance of *S*. *aureus*. The bronchoalveolar lavage fluid (BALF) of CCR2-DTR mice after *S*. *aureus* infection displayed significant decreases in interleukin-12 (IL-12), IFN-γ, IP-10, MIG and RANTES, all IFN-γ regulated, compared to wild-type (WT) infected controls. NK cells were identified as the main producers of IFN-γ, but both NK cells and IFN-γ were dispensable for clearance. We demonstrated through cytokine production and RNA-seq analysis that IL-12 and IL-12 regulated genes are strongly induced in monocytes upon *S*. *aureus* infection. Administration of IL-12 during infection restored the bacterial burdens in the BALF and lungs of monocyte-depleted CCR2-DTR mice to the levels of WT mice, independent of IFN-γ. In the absence of monocytes, alveolar macrophages are the primary phagocytic cells, and IL-12 influences their capacity to produce reactive oxygen species and clear *S*. *aureus*. These results show that production of IL-12 contributes to the control of *S*. *aureus* via its influence on alveolar macrophage function.

## Author summary

*Staphylococcus aureus* is an important cause of infections across numerous bodily sites. One area of significant disease is the respiratory tract, leading to pneumonia. The monocyte is a cell of the immune system that is recruited to the airway during infection. The full role this cell plays during *S*. *aureus* infection is not completely understood. We show here that it is important for clearance of *S*. *aureus* from the airway. We also demonstrate that its role is not in direct bacterial clearance but through effects exerted upon another cell, the alveolar macrophage. We show that monocytes secrete important signaling molecules, including one, IL-12, that impacts the capacity of the alveolar macrophage kill *S*. *aureus*. This work demonstrates that different cells of the immune system can accentuate the antimicrobial ability of other cell types during infection.

## Introduction

*Staphylococcus aureus* is a major bacterial pathogen that can cause a variety of diseases including pneumonia. Due to its high propensity to develop antibiotic resistance, treatment options against this pathogen have become scarce and, as a result, methicillin-resistant *Staphylococcus aureus* (MRSA) alone was associated with 748,000 deaths globally in 2019 and over $1.7 billion in healthcare costs in the United States [1]. To this day, no vaccine against *S*. *aureus* is available. A better understanding of the immune responses to this pathogen could allow the design of more efficacious treatments that rely on the immune system and could act together with antibiotics.

Monocytes are large mononuclear cells that represent 4% and 10% of the white blood cells in mice and humans, respectively [2]. They are derived from hematological precursors in the bone marrow. Their egress from this compartment and subsequent recruitment to peripheral tissues is highly dependent on the expression of C–C motif chemokine receptor 2 (CCR2) [3]. The most renowned feature of monocytes is their ability to differentiate into macrophages during infection and inflammation. Previous studies have shown that alveolar macrophages are important innate mediators of clearance of *S*. *aureus* from the airways. In models of *S*. *aureus* acute pulmonary infections, depletion of alveolar macrophages in mice led to increased mortality rates and bacterial burdens [4,5].

Monocytes are no longer viewed just as precursors for macrophages and publications from several groups have highlighted the heterogeneity in the response of alveolar macrophages and recruited monocytes against diverse pathogens in the airway. In a model of pulmonary fungi infection with *Aspergillus fumigatu*s, recruited monocytes were found to perform essential direct and indirect functions that contribute to innate antifungal immunity [6]. However, in the same model, alveolar macrophages, despite their participation in innate defense and their demonstrated in vitro and in vivo fungicidal effects, appeared to be dispensable for clearance of the pathogen [7–9]. In a model of infection with *Mycobacterium marinum*, monocytes and alveolar macrophages had drastically distinct behaviors, where alveolar macrophages were microbicidal and recruited monocytes permissive to infection [10].

IL-12 functions across multiple cell types. The expression of the two subunits IL-12p35 and IL-20p40 is required for the secretion of the active, IL-12p70 cytokine [11,12]. IL-12 is produced by a variety of cell types, with most production derived from antigen presenting cells. Myeloid derived cells [13], such as monocytes produce IL-12 in response to different stimuli, including *S*. *aureus* [14–16]. The initial role of IL-12 was discovered in the maturation of T lymphocytes and activation of NK cells [13,17,18]. IL-12 can also regulate cellular pathways important for the function of the immune system, such as through the activation of IFN-γ [19–22]. IL-12 is recognized by its receptor, composed of IL12Rβ1 and IL12Rβ2 [23]. Activation of the IL-12 receptor occurs through Janus kinase and signal transducers and activators of transcription (JAK-STAT) family members [24]. IL-12 signaling utilizes pSTAT4 [25]. The IL-12 receptor is highly expressed on lymphocytes and NK cells but is also expressed on macrophages [26]. IL-12 has been shown to influence the function of macrophages through: chemotaxis, peptide presentation, nitric oxide production, growth and bactericidal activity [26–31].

Monocytes are plastic antigen presenting cells with a demonstrated role in both pro- and anti-inflammatory systems. Little is known about the role of these cells in clearance of *S*. *aureus* from the airway and, as monocytes are one of the most abundant antigen-presenting cell populations during inflammation, elucidating their roles and regulation in this context is of great interest. Here, using a transgenic mouse model of acute monocyte depletion, we demonstrate that these cells play a role in bacterial clearance of *S*. *aureus* from the lung. We identified that IL-12 was a cytokine significantly reduced in the absence of monocytes. An interleukin-12 (IL-12) signaling gene signature was identified in lung monocytes from *S*. *aureus* infected mice and we confirmed the production of this cytokine in stimulated cells. Moreover, IL-12 administration at the time of infection in mice lacking monocytes reduced their bacterial burden to WT levels in the absence of IFN-γ. IL-12 was subsequently found to influence alveolar macrophage (AM) function. Altogether, these findings show that production of IL-12 by monocytes contributes to the control of *S*. *aureus* pulmonary infection.

## Results

### Monocytes are important for clearance of *S*. *aureus* from the airway

**Fig 1:**
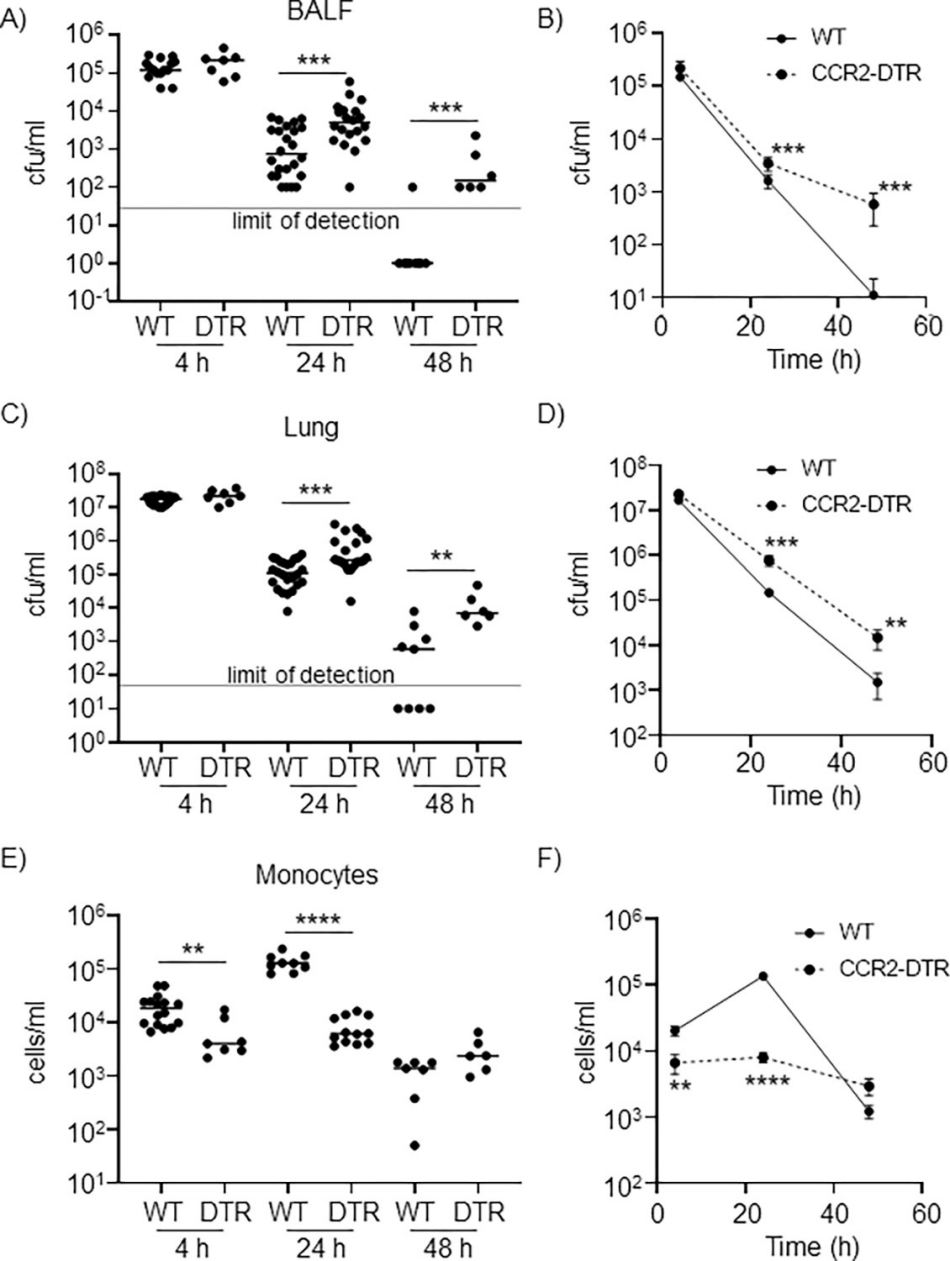
Monocytes are important for airway clearance of *S*. *aureus*. Wild-type and CCR2-DTR mice were injected intraperitoneally with diphtheria toxin. 24 h later, mice were inoculated intranasally with 3 x 10^7^ CFU of *S*. *aureus* USA300. Mice were sacrificed at the times indicated. BALF and lung homogenates were enumerated for bacterial counts (A-D) and monocytes were quantified in BALF (E&F). A, C, E show individual data, while B, D and F showed average values charted over time. 4 h: WT, n = 15, CCR2-DTR, n = 7, 24 h: WT, n = 24, CCR2-DTR, n = 20, 48 h: WT, n = 9, CCR2-DTR, n = 6. Each dot represents a mouse. Lines display median. DTR = CCR2-DTR. Line graphs show mean and SEM. Mann-Whitney statistical tests were used. Data is from at least two independent experiments. ****p<0.0001, ***p<0.001 and **p<0.01.

To determine the role of monocytes in clearance of *S*. *aureus* from the airway, we utilized CCR2-DTR transgenic mice in which the diphtheria toxin receptor (DTR) is expressed under the control of the CCR2 promoter. Administration of diphtheria toxin (DT) to these mice depletes monocytes from the lung and the periphery. CCR2-depleted mice and WT controls were infected intranasally with *S*. *aureus*. Early after infection, no significant differences in bacterial burden were observed between the airways of WT and CCR2-depleted mice (**Fig 1A–1D**). However, starting at 24 hours post infection, a significant increase in bacterial burden (3.7-fold in BALF and 3.6-fold difference in lung; p<0.001) was observed in CCR2-depleted mice compared to WT mice (**Fig 1A–1D**). By 48 hours after inoculation, the infection was almost resolved in WT mice, whereas bacterial burdens were still significantly higher in CCR2-depleted mice (51.5-fold difference in BALF, p<0.001; 8.8-fold difference in lung, p<0.01) (**Fig 1A–1D**). When graphed continuously, over time CCR2-DTR mice fair much poorer in their capacity to clear the infection (**Fig 1B and 1D**). Monocyte depletion was confirmed by flow cytometry analysis (**Fig 1E and 1F**). In CCR2-depleted mice, lung monocyte concentrations remained consistently low throughout the infection process, with less than 10,000 cells/mL at each time point (**Fig 1F**). By 24 h we observed a 94% (p<0.0001; **Fig 1E and 1F**) decrease in lung monocytes in the CCR2-DTR mice, comparable to other studies [32, 33]. In WT mice, however, a significant monocytic influx was observed in the lung 4 h after the beginning of infection with a peak of over 100,000 cells/mL detected 24 h post inoculation. Following the infection timeline, as the bacterial burden decreased in WT mice, monocyte numbers also did, with about 1,000 cells/mL detected 48 h post inoculation (**Fig 1F**). We also evaluated the effects of CCR2 depletion on other cell types, by quantifying the main innate immune cell subsets in the BALF of WT and CCR2-depleted mice, 24 h post infection (**S1 Fig**). Eosinophils were significantly decreased in CCR2-depleted mice (1.87-fold, p<0.05) but none of the other cell subsets appeared to be affected by the depletion. These data suggest that monocytes are required for effective clearance of MRSA pulmonary infection, as mice lacking monocytes exhibit increased bacterial burdens and delayed clearance.

**Fig 2:**
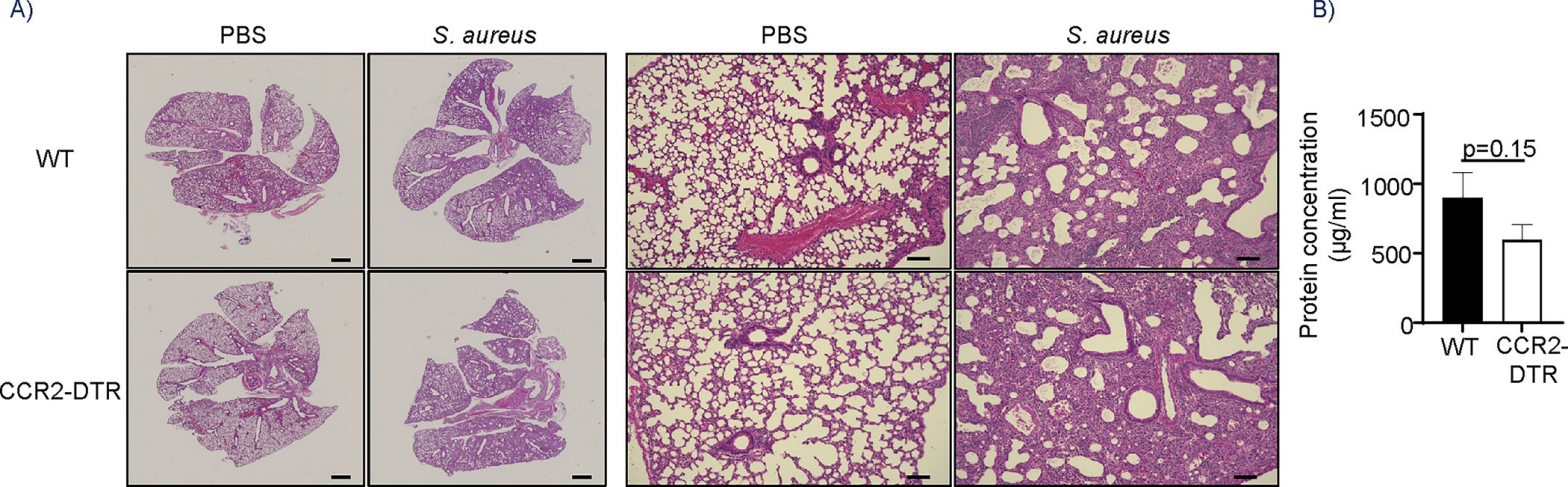
Monocyte depletion does not influence pulmonary pathology after *S*. *aureus* infection. WT and CCR2-DTR mice were injected with DT and inoculated with either PBS or *S*. *aureus* intranasally 24 h later. A) Representative hematoxylin and eosin-stained lung sections 24 h post infection (scale bar 1mm–left panel; 100 μm–right panel). B) Average total protein content in the BALF of WT and CCR2-depleted mice 24 h post infection. WT, n = 10; CCR2-DTR, n = 12. Graphs show mean and SEM. An unpaired t-test was used.

Increased bacterial burdens are commonly accompanied by poor lung pathology. However, in this context, histological examination of lung sections did not reveal any significant differences in lung pathology between WT and CCR2-depleted mice. Both models exhibited similar loss of alveolar structure and cellular infiltrates (**Fig 2A**). We also did not observe any significant differences in total protein content in the BALF of WT and CCR2-depleted mice, suggesting similar levels of lung injury between groups (**Fig 2B**).

### IFN-γ regulated cytokines are reduced in the absence of monocytes

**Fig 3:**
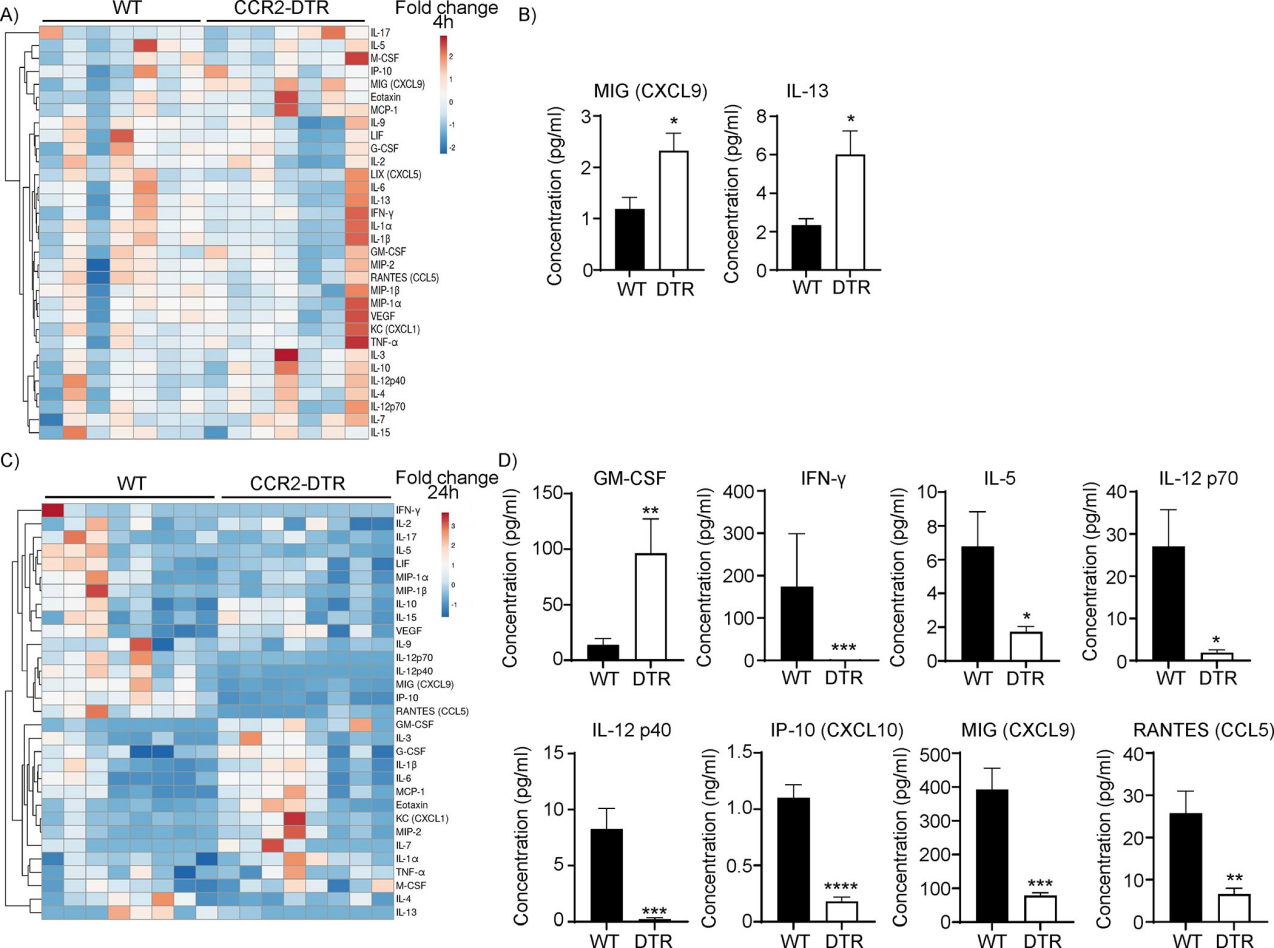
Monocytes contribute to cytokine production in the airway. WT and CCR2-DTR mice were injected with diphtheria toxin 24 h prior to intranasal *S*. *aureus* infection. BALF was collected and cytokines quantified A&B) 4 h and C&D) 24 h after infection. A&C) Heatmap representation of all cytokines quantified. Unit variance scaling is applied to rows. Rows are clustered using correlation distance and average linkage. B&D) Graphs displaying significant differences for each time point. DTR = CCR2-DTR. Graphs display mean with SEM. B) An unpaired t-test was used. D) For IP-10 and MIG an unpaired t-test was used, all other comparisons used a Mann-Whitney test. A&B) n = 7. C&D) n = 8. ****p<0.0001, ***p<0.001, **p<0.01 and *p<0.05.

To determine the effects of monocyte depletion on cytokine production during *S*. *aureus* respiratory infection, we quantified cytokines and chemokines in the BALF of WT and CCR2-depleted mice, 4 h and 24 h post intranasal infection. Monocyte depletion had negligible effect on cytokine production 4 h post infection, with only MIG (CXCL9) and IL-13, significantly altered in the BALF of CCR2-depleted mice compared to WT, out of the 32 quantified secreted factors (**Fig 3A and 3B)**. At 24 h post infection, CCR2-depleted mice, despite possessing increased bacterial burdens, commonly accompanied with increased inflammation and cytokine release, displayed several cytokines with significantly lower levels compared to the control group (**Fig 3C and 3D**). The cytokines IFN-γ, IL-12p40, IL-12p70, IP-10, MIG and RANTES were all significantly decreased in the absence of monocytes. GM-CSF was the only significantly increased cytokine in the BALF of the CCR2-depleted mice (**Fig 3D**). All these decreased cytokines have been shown to be directly or indirectly regulated by IFN-γ [21, 34–36]. These results indicate that monocytes participate in the cytokine response to *S*. *aureus* pulmonary infection.

### IFN-γ is dispensable for *S*. *aureus* clearance in the airway

**Fig 4:**
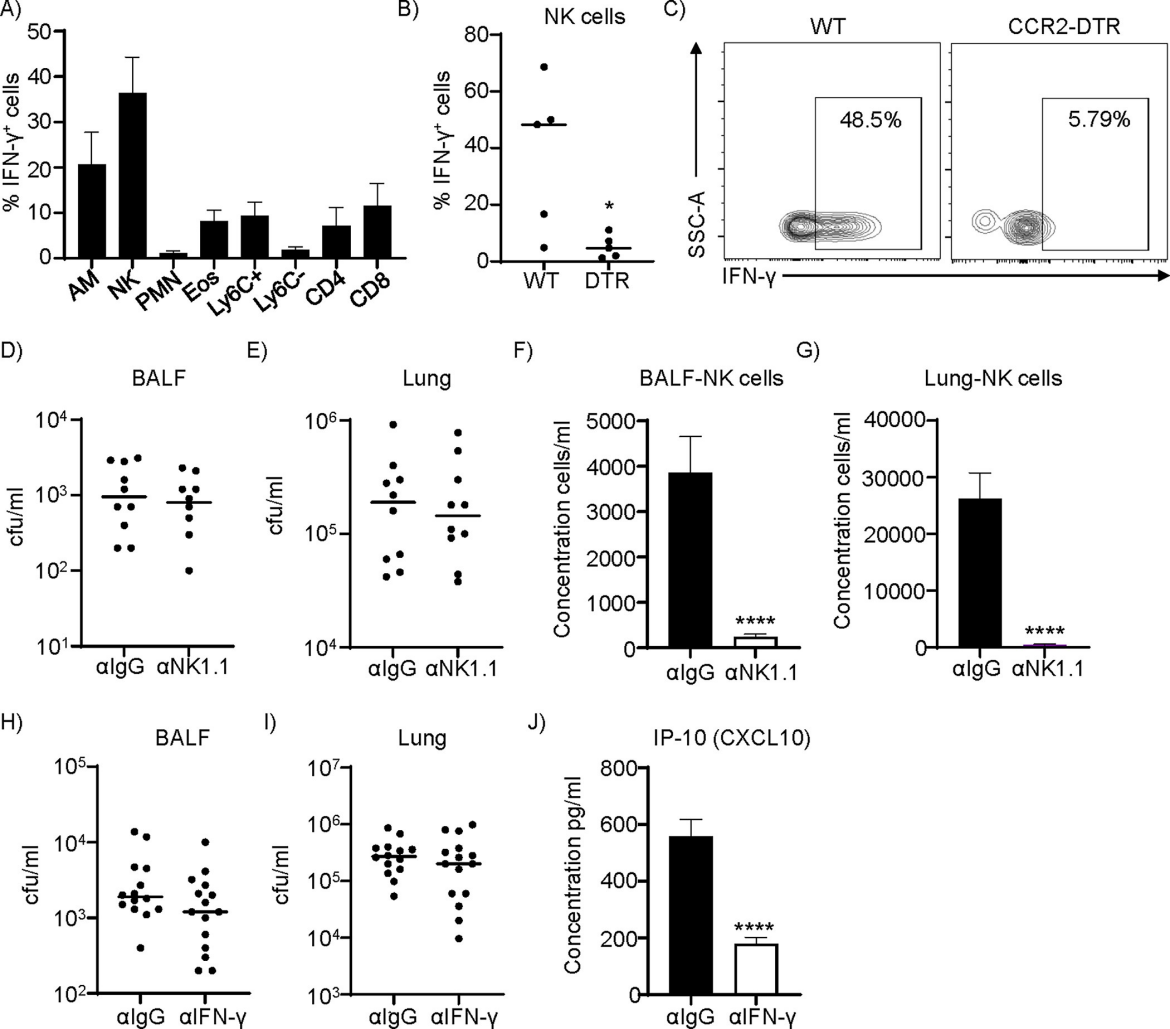
NK cells and IFN-γ are dispensable for *S*. *aureus* respiratory infection. A) WT mice were inoculated intranasally with *S*. *aureus*. BALF was collected 24 hours post infection and intracellular IFN-γ production was detected in the main immune cellular subsets by flow cytometry. n = 7. B) IFN-γ positive NK cells in the BALF of WT or CCR2-DTR mice after monocyte depletion. n = 5. C) Representative contour plot of IFN-γ staining in NK cells, gated on CD45^+^NK1.1^+^. WT mice received two injections of either NK1.1 antibody or control IgG at days 3 and 1 before *S*. *aureus* infection. BALF and lung homogenates were collected 24 h later and analyzed for D&E) bacterial counts and F&G) NK cell numbers. n = 10. WT mice received IFN-γ neutralizing antibody or control IgG simultaneously to *S*. *aureus* intranasal infection. H) BALF and I) lung were quantified for bacterial counts. n = 14 for αIgG, n = 15 for αIFN-γ. J) BALF IP-10 ELISA (CXCL10) quantification. n = 18. DTR = CCR2-DTR. An unpaired t-test was used in B), D) and Mann-Whitney test in E), F), G), H), I) and J). AM-alveolar macrophage, NK-natural killer, PMN-polymorphonuclear, neutrophils, Eos-eosinophil, Ly6C^+^-Ly6C positive monocytes, Ly6C^—^Ly6C negative monocytes, CD4-CD4 T cells and CD8-CD8 T cells. Each point represents a mouse. Lines display median. Graphs show means with SEM.

As IFN-γ and IFN-γ-related cytokines were significantly reduced in infected CCR2-depleted mice (**Fig 3D**), we hypothesized that IFN-γ could contribute to bacterial clearance in our model. We first sought to identify the main cell type responsible for its production in our infection model. To do so, we infected WT mice intranasally with *S*. *aureus* and quantified IFN-γ production in the major immune cell subsets by intracellular staining followed by flow cytometry. We found that NK cells had the highest frequency of IFN-γ^+^ cells in these conditions with over 36.45% of these cells generating IFN-γ (**Fig 4A**). We also found that the percentage of IFN-γ-producing NK cells was significantly (p<0.05) reduced in CCR2-depleted mice (**Fig 4B and 4C**). A subset of NK cells has also been shown to express CCR2 [37, 38]. To determine that our CCR2-DTR phenotype was not due to loss of NK cells (although we did not see changes in their numbers, **S1 Fig**) or that their production of IFN-γ was involved in protection, we depleted NK cells using neutralizing antibodies. Mice lacking NK cells did not exhibit significant differences in pulmonary bacterial burdens (**Fig 4D and 4E**), despite showing efficient depletion of NK cells (**Fig 4F and 4G**). This suggested that IFN-γ production by NK cells is dispensable for *S*. *aureus* clearance. We then evaluated whether IFN-γ was necessary, again using antibody neutralization. Neutralization of IFN-γ did not alter bacterial clearance compared to that of mice receiving control IgG (**Fig 4H and 4I**). Efficiency of IFN-γ neutralization was confirmed by the significant reduction in IP-10/CXCL10, an IFN-γ-regulated cytokine, in mice that received IFN-γ-neutralizing antibodies (**Fig 4J**). These data show that both NK cells and IFN-γ are dispensable for *S*. *aureus* pulmonary clearance.

### Monocytes produce IL-12 in response to *S*. *aureus*

**Fig 5:**
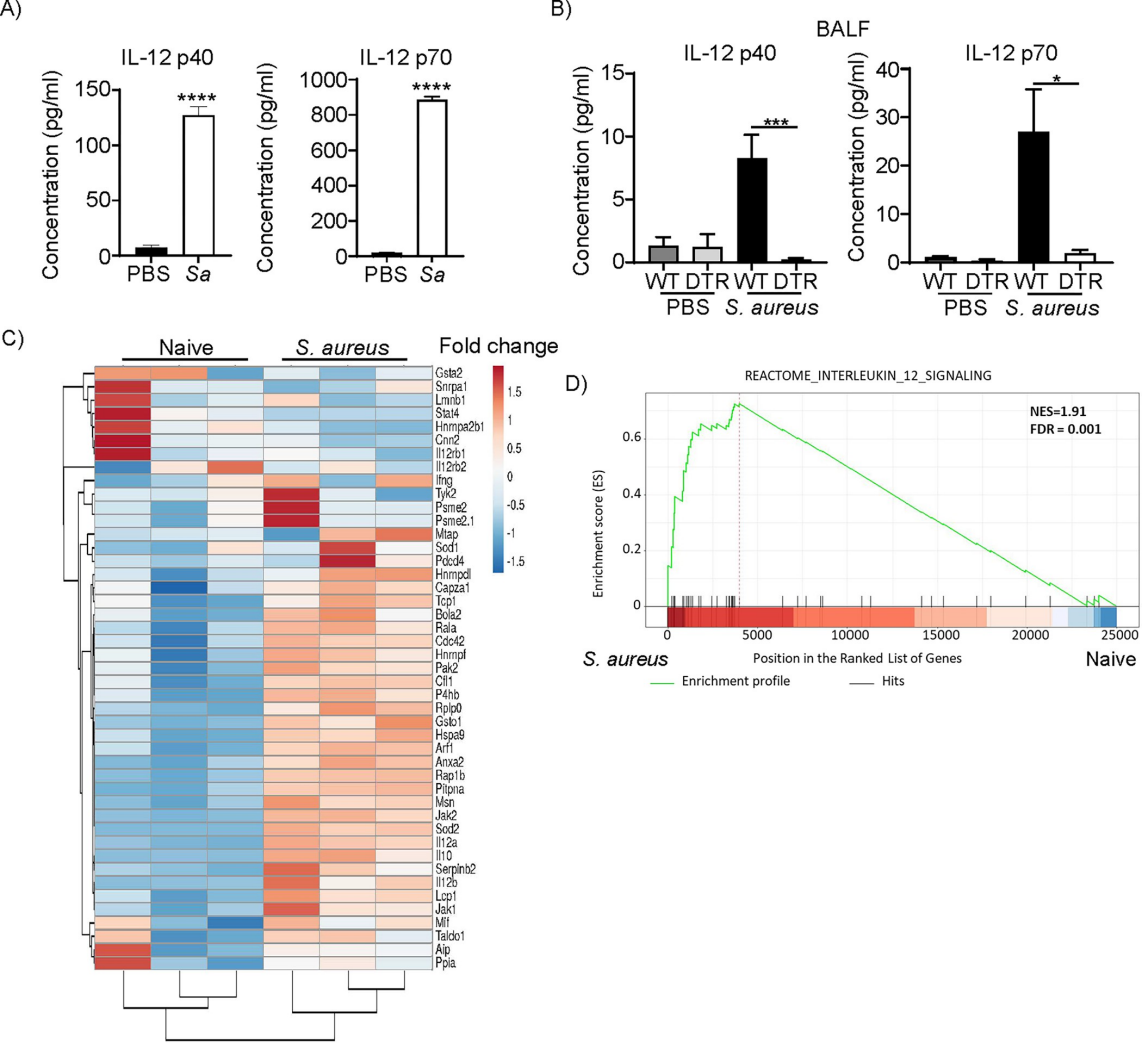
Monocytes produce IL-12 in response to *S*. *aureus*. A) Bone marrow monocytes were stimulated with PBS or *S*. *aureus* (*Sa*) for 24 h and IL-12 produced was quantified by ELISA. PBS n = 6, *S*. *aureus* n = 5. B) Levels of IL-12 from BALF of mice 24 h after infection. Naive mice, n = 2, infected, n = 8. A Mann Whitney test was used. Bone marrow monocytes from naïve mice and monocytes from the airways of *S*. *aureus* infected mice were sort purified and transcripts analyzed by RNA-seq. C) Heat map showing expression levels of IL-12 signaling pathway genes. Unit variance scaling is applied to rows. Rows are clustered using correlation distance and average linkage. n = 3. D) GSEA analysis of Reactome IL-12 signaling. NES-normalized enrichment score. FDR-false discovery rate. Bar graphs show mean and SEM. ****p<0.0001.

Having demonstrated that IFN-γ does not play a role in clearance of *S*. *aureus* in our model of infection we went back to our cytokine data. IL-12 can also regulate production of IFN-γ [20–22] so we sought to determine if monocytes were responsible for its production. To determine if monocytes produce IL-12 in response to *S*. *aureus*, we incubated monocytes from naïve mice with live *S*. *aureus* for 16 h and quantified IL-12 in the supernatant by ELISA. We found this cytokine to be significantly increased in monocytes stimulated with *S*. *aureus* compared to unstimulated cells (**Fig 5A**). IL-12 was induced in vivo after *S*. *aureus* infection and was significantly decreased in the absence of monocytes (**Fig 5B**). To further investigate the products generated by monocytes in response to *S*. *aureus* we examined their response by RNA-seq. The gene expression of purified monocytes from *S*. *aureus* infected airways were compared to naïve monocytes from the bone marrow, due to the low number present in naïve airways. We identified 2,161 differentially expressed genes that exhibited changes greater than 4-fold (p<0.05). In infected cells, the majority of the upregulated canonical pathways were related to pathogen response and cell maturation (**S2 Fig**). As expected, most of the top upregulated genes were predicted to encode secreted and cytoplasmic factors such as cytokines (**S1 Table**) and many of the downregulated molecules were nuclear factors linked with kinetochore metaphase signaling and cell cycle control of chromosomal replication (**S2 Table**). We then examined the IL-12 response more closely. Using the reactome gene set for IL-12 signaling, we observed that 38 of the 46 genes in this pathway were upregulated in monocytes after infection (**Fig 5B**). Gene set enrichment analysis (GSEA) showed a significant (FDR = 0.001) enrichment for IL-12 signaling in infected monocytes (**Fig 5C)**. These results demonstrate that monocytes can make IL-12 in response to *S*. *aureus* infection.

### IL-12 restores bacterial clearance in the absence of monocytes

**Fig 6:**
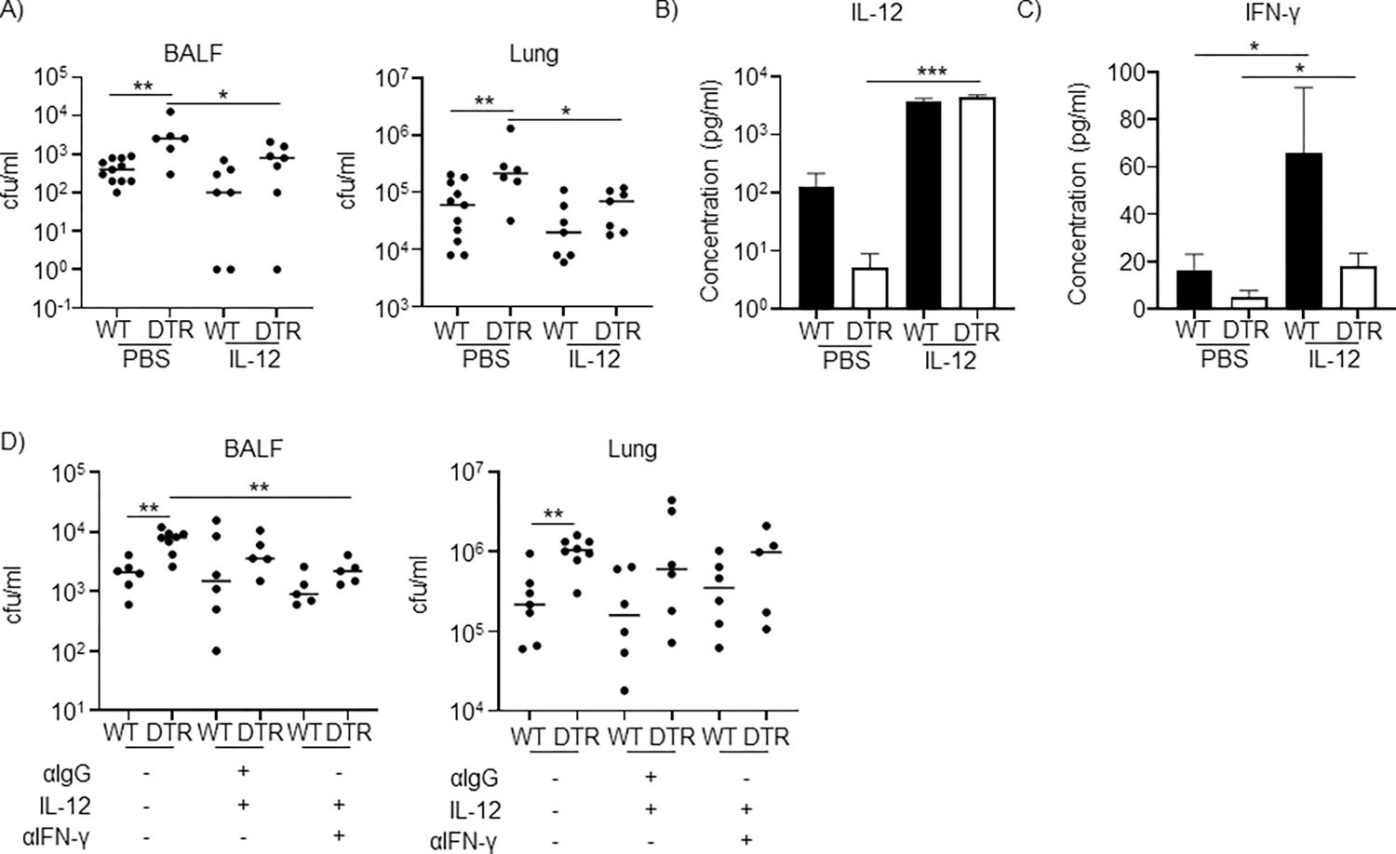
IL-12 restores bacterial clearance in monocyte depleted mice, independently of IFN-γ. WT and CCR2-depleted mice were inoculated intranasally with either *S*. *aureus* or *S*. *aureus* along with IL-12. A) BALF and lung homogenates were enumerated for bacterial counts 24 hours later. B) BALF IL-12p70 concentrations were quantified by ELISA. WT PBS n = 8, DTR PBS n = 7, WT+IL-12 n = 4 and DTR+IL-12 n = 8. C) IFN-γ concentrations in BALF were quantified by ELISA. WT PBS n = 8, DTR PBS n = 7, WT+IL-12 n = 5 and DTR+IL-12 n = 7. WT and CCR2-depleted mice received IFN-γ neutralizing antibody or control IgG one day before intranasal *S*. *aureus* infection and IL-12 administration. D) BALF and lung homogenates were quantified for bacteria 24 h later. Comparisons were assessed using unpaired t-tests in C)-WT and D-BALF, and Mann-Whitney tests in A), B), C)-CCR2-DTR and D)-lung base on normality tests. WT PBS n = 7, DTR PBS n = 8, WT+IL-12+IgG n = 6, DTR+IL-12+IgG n = 6, WT+IL-12+αIFN-γ n = 6 and DTR+IL-12+αIFN-γ n = 5. DTR = CCR2-DTR. Lines display median. Bar graphs show mean and SEM. Statistically insignificant differences are not labelled. **p<0.01 and *p<0.05.

As we observed IL-12 to be significantly reduced in CCR2-DTR mice (**Fig 3D**) and monocytes can produce it (**Fig 5**), we hypothesized this cytokine defect could be responsible for the increased bacterial burdens observed in monocyte-depleted mice. To test this, we repeated our 24 h airway infection model in WT and CCR2-depleted mice and simultaneously administered recombinant IL-12 to a subset of the mice. We observed that IL-12 administration did not affect bacterial burdens in WT mice (**Fig 6A;** not significant). However, in CCR2-depleted mice, addition of IL-12 reduced the bacterial burdens to WT levels (**Fig 6A**). CCR2-DTR mice with IL-12 were not statistically different to WT control or WT mice with IL-12. We also confirmed that IL-12 levels were similar in the mice that had received recombinant IL-12 (**Fig 6B**). Both WT and CCR2-DTR mice had similar levels of IL-12 and IL-12 levels were significantly higher in CCR2-DTR mice that received IL-12 compared to non-complemented controls (**Fig 6B**). Consistent with IL-12 being able to induce IFN-γ, both WT and CCR2-DTR mice that had received recombinant IL-12 exhibited significant increases in IFN-γ (**Fig 6C**). After IL-12 administration, CCR2-DTR mice had levels of IFN-γ comparable to WT mice (**Fig 6C**). To rule out the potential effects of IFN-γ in this protection phenotype, we repeated this experiment in mice that had received IFN-γ-neutralizing antibody or isotype control. As before, we observed a significant difference between WT and CCR2-DTR mice under normal conditions (**Fig 6D**). We observed that treatment with IL-12 in the presence of neutralizing IFN-γ antibody still reduced bacterial burdens in CCR2-DTR mice compared to the controls (**Fig 6D**; p<0.01). We did not observe the same difference in the lung due to data variation, however, in the presence of IL-12, there were no differences between WT and CCR2-DTR mice in the BALF or the lung tissue (**Fig 6D**). These results indicate that IL-12 complementation was sufficient to rescue the defect in bacterial clearance observed in CCR2-depleted mice and that this IL-12 protection appears to be mediated in an IFN-γ-independent manner.

### IL-12 drives killing capacity of alveolar macrophages

**Fig 7:**
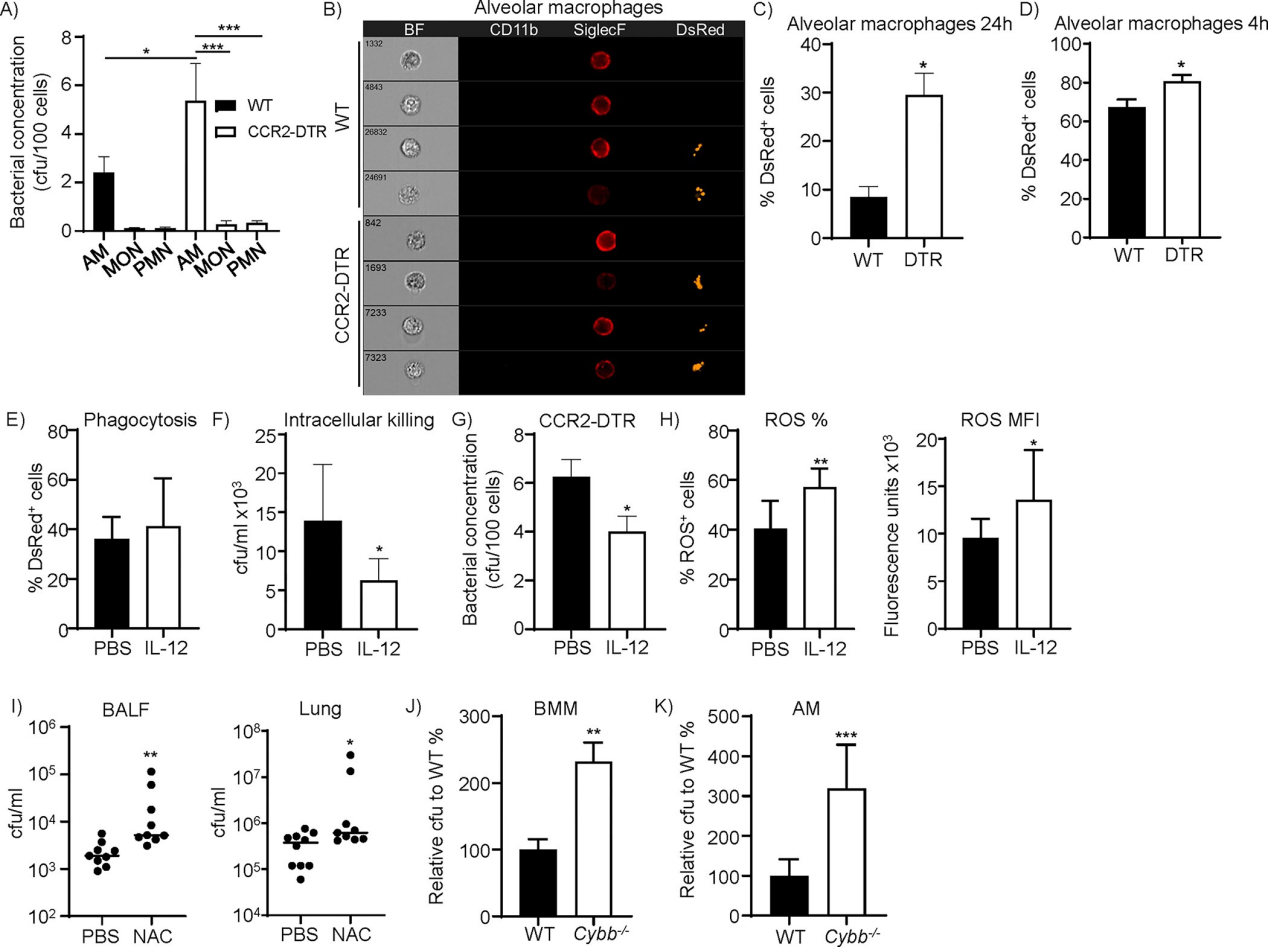
IL-12 potentiates alveolar macrophage killing. A) Mice were infected intranasally with *S*. *aureus* strain USA300 24 h after monocyte depletion. Lungs were harvested 24 hours later, and alveolar macrophages, neutrophils and monocytes were collected by fluorescence activated cell sorting. Individual cell types were plated on LB agar plates to enumerate bacterial numbers. n = 6. B) BALF was collected 24 h after infection using DsRed *S*. *aureus* and analyzed by Imagestream flow cytometry. Representative data for alveolar macrophages. C) Quantitative alveolar macrophage data. n = 3. WT and CCR2-DTR mice were given diphtheria toxin 24 h prior to intranasal infection with DsRed-expressing *S*. *aureus*. D) BALF was collected 4 h later for flow cytometry analysis to quantify bacteria inside immune cells. WT n = 5, CCR2-DTR n = 4. E) Phagocytosis of AM one hour after *S*. *aureus* incubation. WT-n = 12, DTR-n = 9. F) Intracellular killing of AM in the presence and absence of IL-12. n = 6. G) Bacterial counts in AM from CCR2-DTR mice infected with *S*. *aureus* for 24 h with or without exogenous IL-12 treatment. No IL-12 n = 7, IL-12 n = 8. H) ROS generation of AM in the presence of *S*. *aureus* with and without IL-12 stimulation. n = 11. I) Mice were treated with N-acetylcysteine or vehicle control the day prior to *S*. *aureus* intranasal challenge. BALF-WT and NAC n = 9, lung WT n = 10 and NAC n = 9. J) BMM and K) AM from WT and *Cybb*^-/-^ mice were infected with *S*. *aureus* before gentamicin treated to quantify intracellular killing. In J) n = 8 and K) WT n = 8 and *Cybb*^-/-^ n = 7. Each dot represents a mouse. Lines display median. DTR = CCR2-DTR, AM-alveolar macrophages, PMN-polymorphonuclear cells, neutrophils, MON-monocytes. Graphs show means with SEM. Data in A) was tested with an ANOVA and Tukey’s multiple comparison test. C-H) used an unpaired t-test, and I-K) a Mann Whitney test. ****p<0.0001, ***p<0.001, **p<0.01 and *p<0.05.

To gain an idea on the cells important for clearance in the presence and absence of monocytes, we developed an assay to assess bacterial processing by individual cell types. In this experiment, WT and CCR2-depleted mice were infected intranasally with *S*. *aureus* and, 24 h post infection, alveolar macrophages (AM), neutrophils and monocytes were collected from the lungs by fluorescence activated cell sorting. The individual cell types were serially diluted and plated onto agar plates to quantify internalized bacteria. The number of live bacteria per cell was increased in AM from CCR2-DTR mice compared to WT mice, and AM had the most bacteria amongst the cell types examined (**Fig 7A**). These data suggested that AM were able to phagocytose the most bacteria on a per cell basis and bear the burden of increased bacterial load in the absence of monocytes or had a reduced ability to kill the internalized bacteria. Only a small portion of the monocytes from WT mice were found to take up bacteria (**Fig 7A**), suggesting that monocytes were potentially influencing AM function.

We further investigated the increased bacterial numbers observed in AM by using ImageStream flow cytometry analysis to track bacteria inside the cells 24 h post infection. ImageStream is a technology that integrates the features of flow cytometry and immunofluorescence. To utilize Imagestream, we inoculated WT and CCR2-depleted mice with an *S*. *aureus* strain that expresses DsRed. In CCR2-depleted mice, we observed that bacteria were indeed inside AM (**Fig 7B**) and a significant increase was quantified inside AM (**Fig 7C**). We extended our Imagestream analysis and showed that even at 4 h after infection we observed that AM had an increase in bacteria in the absence of monocytes (**Fig 7D**).

The increase in bacterial burden observed in CCR2-depleted mice could be linked to heightened uptake or an inability to kill *S*. *aureus*. Our data also suggests that IL-12 drives our clearance phenotype. To determine if phagocytosis was impacted, we cultured AM isolated from mice in the presence and absence of IL-12 and quantified bacterial uptake one hour after incubation. At this early time point we did not observe a difference, suggesting that phagocytosis was not impacted by IL-12 (**Fig 7E**).

As phagocytosis was not impacted, we next attempted to determine if IL-12 influences the ability of AM to kill *S*. *aureus*. To do this we examined the ability of AM to kill intracellular *S*. *aureus* in the presence of absence of IL-12. In the presence of IL-12, AM reduced intracellular *S*. *aureus* numbers by over 50% (P<0.05; **Fig 7F**). We further demonstrated this effect in vivo. CCR2-DTR mice were infected with *S*. *aureus* with or without IL-12 administration and 24 h later AM were purified out of the lungs and bacterial numbers determined. We observed that with IL-12 there were significantly less bacteria present inside AM from CCR2-DTR compared to untreated controls (**Fig 7G**).

A major mechanism of AM killing is the production of reactive oxygen species (ROS). Therefore, we quantified reactive oxygen species (ROS) activity in AM. ROS activity was detected to be higher in AM with IL-12 in response to *S*. *aureus* compared to the controls (**Fig 7H**). We confirmed the importance of ROS during infection by pre-treating mice with the ROS scavenger N-acetylcysteine and observed that mice had significantly higher levels of bacteria with ROS inhibition (**Fig 7I**). We further showed that ROS was important for macrophage killing using mice lacking *Cybb* (gp91phox^-^). We first demonstrated using bone marrow derived macrophages that without ROS there was a reduced ability to kill *S*. *aureus* inside the cells (**Fig 7J**). This observation was then confirmed with primary AM, which in the absence of ROS had significantly higher numbers of *S*. *aureus* inside (**Fig 7K**). These data suggest that monocytes aid in the clearance of *S*. *aureus* through production of IL-12 that increases the capacity of AM to kill through enhanced ROS activity.

## Discussion

We have shown that upon depletion of monocytes in a model of *S*. *aureus*-mediated pneumonia, delayed bacterial clearance from the lung tissue and airways occurs. Our data showed that only a small portion of the bacteria were localized inside the monocytes at the time of infection. Our results suggest that the contribution of monocytes to bacterial clearance in this context is not dependent on phagocytosis or direct killing. Meanwhile, the absence of monocytes led to an increase in the number of *S*. *aureus* in AM. This increase was influenced by IL-12 altering ROS levels and bactericidal function of AM. Monocytes have previously been shown to display decreased phagocytic and microbicidal properties against *S*. *aureus* in vitro [39–41]. Intrinsic cellular differences may also influence phagocytosis by these cell types since, in the presence of high bacterial burdens, the ability of monocytes to uptake opsonized bacteria quickly became saturated whereas that of neutrophils and AM remained stable [42]. We did not observe monocytes to play a significant role in direct *S*. *aureus* clearance.

We observed that both NK cell depletion and IFN-γ neutralization did not alter bacterial clearance in our WT pneumonia model and that IL-12, independently of its effect on IFN-γ, was able to rescue the delayed bacterial clearance observed in the CCR2-depleted mice. We demonstrated that NK cells were the main producers of IFN-γ and they are known to be affected by IL-12 [31,43]. Both CXCL9 and RANTES are gamma regulated cytokines, so we expect their levels to be directly affected by the concomitant decrease in IFN-γ [21,34–36]. IL-12 has previously been shown to contribute to protection against pulmonary infection with MRSA. This is also despite the fact that the levels of IL-12 in WT infected mice are not high. A study by Nguyen et al. showed that stimulation of IFN-γ-expressing NK cells and downstream IFN-γ signaling in macrophages was the mechanism by which IL-12 conferred protection [31]. In their model of MRSA pneumonia, antibody-mediated depletion of NK cells resulted in significantly increased pulmonary bacterial burdens. Differences between these studies could be explained by differences in inoculum. The prior study used a much higher inoculum, which may involve or evoke different responses. They also observed that IL-12 administration in WT mice resulted in a significant reduction in bacterial burden. Despite using the same dose of cytokine, in our study, IL-12 administration only had a negligeable effect on the respiratory bacterial burdens of WT mice. The experimental model used by Nguyen et al. was prophylactic, since IL-12 was administered 24 hours before infection and resulted in early recruitment of neutrophils and NK cells to the site of infection. In our study, IL-12 was administered at the time of infection to more closely mimic the WT cytokine milieu at the peak of infection.

The impaired clearance in CCR2-DTR mice contrasts to other studies utilizing CCR2 knockout mice. In two studies, the ability of CCR2 knockout mice to clear *S*. *aureus* pneumonia was found to be similar to that of WT mice [44,45]. *Ccr2*^-/-^ mice exhibit constitutive monocytopenia throughout their lives, whereas CCR2-DTR mice only undergo acute monocytic depletion. Although no developmental defect has been observed in *Ccr2*^-/-^ mice, their immune responses could be altered by undetected compensatory mechanisms. The advantage of the temporal depletion mediated by the DTR system is that it will not affect the development of the mice. This could be a reason for the differences between studies. As an example, in a murine post-influenza model of infection, *Ccr2*^-/-^ mice exhibited significantly higher survival and improved bacterial clearance post MRSA challenge [45]. This is also a co-infection model of infection. Prior infection with influenza alters the hosts innate immune system, thus what is required for protection against a primary *S*. *aureus* pneumonia could vary significantly to one that is secondary to a viral infection. It is possible that a prior interaction with influenza alters the response of monocytes to subsequent infections.

IL-12 production by monocytes has been observed in different infection models [15,46,47]. Here, we confirmed by RNA-seq and ELISA that monocytes recruited to the lung can produce IL-12 transcripts in response to *S*. *aureus* infection. A limitation was the comparison of recruited monocytes to bone marrow monocytes, since numbers present at steady-state are not sufficient for experimental analysis. We were also unable to demonstrate that the specific production of IL-12 from monocytes was the driving factor, leaving open the prospect of another cell producing IL-12 through monocyte regulation. Moreover, administration of recombinant IL-12 was able to rescue the defect in bacterial clearance observed in CCR2-depleted mice. We also found that this complementation was unaltered by IFN-γ neutralization. This was an unexpected observation since the majority of the immunological functions mediated by IL-12 have been reported to occur through IFN-γ production of activated NK and Th1 cells [48]. However, IFN-γ-independent effects of IL-12 have been observed in infectious diseases models with different types of pathogens including *Aspergillus fumigatus*, *Leishmania majo*r, *Leishmania donovani* and *Salmonella enterica* [49–52]. In these studies, the IFN-γ-independent protective effects of IL-12 were mediated via the induction of proliferative responses in T cells, TNF and nitric oxide synthase responses in the liver or an increase in the oxidative microbicidal functions of monocytes via the production of superoxide anion [49–52]. Although there is limited data, there is some evidence to suggest monocytes might be responsive to IL-12 [53]. However, macrophages have also been shown to both express the IL-12 receptor and be responsive to IL-12 [26–30,54]. Our new data seems to best align with this later point, increasing ROS activity to improve clearance *S*. *aureus*. While we demonstrated that inhibition of ROS impacts bacterial clearance and a lack of ROS in AM influences bacteria handling in vitro, we have not demonstrated this directly in vivo.

Several studies in humans point to the importance of IL-12 signaling in combating infection. IL-12 deficiency has been associated with increased risk to infection [55–58], including recurrent pneumonias caused by *S*. *aureus* and *Streptococcus pneumoniae* [59]. The IL-12 antibody therapeutic, Ustekinumab is also associated with increased infections [60–62], while IL-12 receptor beta 1 deficiency has been associated with increased susceptibility to infections by non-tuberculous mycobacteria and *Salmonella* spp [63–65]. There is also data regarding increased infection rates if the downstream signaling (Jak2, Tyk2) is interfered with. Patients on the JAK inhibitors do see increased infection rates and higher rates of pneumonia [66–70]. Mutations in Tyk2 also contribute to patients with hyper-IgE syndrome that suffer from frequent bouts of *S*. *aureus* pneumonia. These reports further support the hypothesis that IL-12 could be important for bacterial clearance.

In summary, we found that depletion of CCR2 positive monocytes in mice led to significant increases in bacterial load in their BALF and lungs 24 to 48 hours post infection with *S*. *aureus*. Monocyte depletion resulted in significant decreases in IL-12, IFN-γ, IP-10, MIG, RANTES and IL-5 in DT-treated CCR2-DTR mice. The production of IL-12 and induction of an IL-12-dependent gene signature were confirmed in purified monocytes. IL-12 administration reduced the bacterial burden of CCR2-depleted mice to WT levels. This effect of IL-12 was unaltered by IFN-γ neutralization and led to increased ROS production and killing by AM. We propose a model whereby monocytes influence the function of AM through production of IL-12 that enhances their antimicrobial capacity through production of ROS. This work adds further depth to the innate immune requirements for combating *S*. *aureus* in the airway.

## Material and methods

### Ethics statement

Animal work in this study was conducted in strict accordance with the recommendations in the Guide for the Care and Use of Laboratory Animals of the NIH (National Academies Press, 2011), the Animal Welfare Act, and US federal law. Protocols were approved by the Institutional Animal Care and Use Committee of Rutgers New Jersey Medical School of Newark, New Jersey, USA.

### Bacterial strains and culture conditions

*S*. *aureus* strain USA300 FPR3757 was grown in Luria-Bertani broth at 37°C to exponential phase (optical density of 1.0 at 600 nm). For the in vivo phagocytosis experiments the *S*. *aureus* strain USA300 JE2 pVT1, expressing DsRed, was grown under the same conditions [71].Alveolar macrophages (AM) were obtained from BALF of mice after red cell lysis. Cells were seeded at 50,000 cells per well of a 96 well microtiter tissue culture treated plate. AM were grown for five days in RPMI 1640 media with 10% heat inactivated fetal bovine serum, penicillin and streptomycin and 500 ng/ml GM-CSF (PeproTech). Intracellular bacterial killing assays involved AM treated with 250 ng/ml of IL-12 (PeproTech) or vehicle control for 24 h prior to incubation with *S*. *aureus* (MOI 1). *S*. *aureus* was incubated with AM for one hour before washing with PBS and incubation with 500 μg/ml of gentamicin to kill extracellular bacteria. Cells were then incubated with 1% triton X-100 and serially diluted to enumerate bacteria.

### Animal studies

Female and male C57BL/6J WT or CCR2-DTR mice [32], 6 to 8 weeks old, were intranasally infected with 3 x 10^7^ colony-forming units (CFUs) of *S*. *aureus* as previously described [72]. Littermates were used as controls. For assessment of bacterial burden, CFUs were enumerated post-infection from bronchoalveolar lavage fluid (BALF) and lung homogenates using serial dilutions on chromogenic media (Becton Dickinson). BALF was also used to quantify cytokine levels using multiplex (mouse 32-plex; Eve Technologies), ELISA (IL-12p40 and p70; Biolegend, IFN-γ; Biolegend, CXCL10; R&D systems) and protein content using Bradford reagent (Bio-Rad). Heatmaps were generated using ClustVis [73].

The effect of ROS on bacterial clearance was tested by administering 100 μg of N-acetylcysteine) per mouse intraperitoneally the day prior to infection. gp91phox^-^ (*Cybb*) mice (from Jackson Laboratories) [74] were used to define the contribution of ROS to macrophage killing of *S*. *aureus*. Bone marrow derived macrophages (BMM) were generated as described [75] before adding exponential phase *S*. *aureus* at MOI 10 for one prior to washing and incubation with 500 μg/ml of gentamicin for 4 h, followed by serial dilution assess bacterial numbers.

For histological examination, lungs were fixed in 4% paraformaldehyde, paraffin embedded and stained with hematoxylin and eosin at the Histology Core Facility (Rutgers NJMS). For depletion of CCR2^+^ cells, CCR2-DTR mice and control littermates received 200 ng of diphtheria toxin intraperitoneally, 24 hours before infection. Efficiency of depletion was assessed by flow cytometry. To deplete NK cells, mice were injected intraperitoneally with 250 μg of anti-mouse NK1.1 (PK136; BioXCell) or with IgG2a isotype control (C1.18.4; BioXCell), 72 and 24 hours before infection. Efficiency of depletion was assessed by flow cytometry. For IFN-γ neutralization experiments, mice received 1 mg/mouse of IFN-γ neutralizing antibody (XMG1.2; BioXCell) or isotype control (HRPN; BioXCell) intraperitoneally, 24 hours before infection. For IL-12 complementation experiments, mice received 1μg/mouse of recombinant IL-12p70 (PeproTech) intranasally at the time of infection.

### Flow cytometry

Cells in BALF and lung homogenates were stained with combinations of the following fluorescently-conjugated antibodies: CD45-AF700 (30-F11), CD103-BV510 (2E7) or CD103-PerCP/Cy5.5 (2E7), Siglec-F-AF647 (E50-2440; BD Biosciences), Ly6C-PE-CF594 (AL-21; BD Biosciences), NK1.1-BV650 (PK136), NK1.1-PE (S17016D), CD11c-BV605 (N418), Ly6G-PerCP/Cy5.5 (1A8) or Ly6G-BV510 (1A8), CD11b-PE-Cy7 (M1/70), MHCII-APC-Cy7 (M5/114.15.2), MARCO-FITC (579511; R&D Systems), CD200R-PE (OX-110), CD86-BV421 (GL-1), CD3-BV650 (17A2), CD4-AF700 (GK1.5) and CD8a-BV421 (53–6.7). Antibodies were purchased from Biolegend unless otherwise stated. Viability was assessed using DAPI. Cells were acquired using a Fortessa X-20 cell analyzer (BD Biosciences). Analysis of flow cytometry data was performed using FlowJoV10 software. Cells in the airway were classified using previously described gating strategies [76] as follows: alveolar macrophages (CD45^+^Ly6C^-^SiglecF^+^CD11c^+^CD11b^-^), neutrophils (CD45^+^SiglecF^-^MHCII^-^Ly6C^+^Ly6G^+^), eosinophils (CD45^+^Ly6C^-^ SiglecF^+^CD11c^-^CD11b^+^), CD11b DC (CD45^+^Ly6C^+^CD11b^+^MHCII^+^CD11c^+^), Ly6C+ monocytes (CD45^+^Ly6C^+^CD11b^+^MHCII^-^Ly6G^-^), Ly6C^-^ monocytes (CD45^+^Ly6C^-^SiglecF^-^CD11b^+^CD11c^+^MHCII^-^), interstitial macrophages (CD45^+^Ly6C^-^SiglecF^-^CD11b^+^CD11c^+^MHCII^+^) and NK cells (CD45^+^NK1.1^+^)

For assessment of interferon-γ production, cells were incubated with brefeldin A (5 μg/mL) and LPS (1 μg/mL) for 2 hours at 37°C, fixed and permeabilized using the FOXP3 Fix/Perm Buffer set (eBioscience) and stained with IFN-γ-PE (XMG1.1) or IgG1, κ isotype control-PE (RTK2071).

Live CD45^+^CD11b^+^MHCII^-^Ly6C^+^Ly6G^-^ monocytes were isolated using a BD FACS ARIA II cell sorter (Flow Cytometry Core facility, NJMS). Cell subsets were sorted from lung single cell suspensions obtained from WT mice challenged 24 hours prior with *S*. *aureus* or from bone marrow single cell suspensions from naïve WT mice. Cells were incubated with either PBS or live *S*. *aureus* at a multiplicity of infection (MOI) of 10 for 16 hours, at 37°C with 5% CO_2_. Cell supernatants were collected, centrifuged at 2,438 x *g* for 5 minutes twice to remove cell pellets and stored at -20°C until IL-12 quantification was performed using the IL-12(p70) mouse ELISA kit (Biolegend), according to the manufacturer’s protocol.

To enumerate the number of *S*. *aureus* in different cell types, WT and CCR2-DTR depleted mice were infected intranasally with *S*. *aureus* for 24 h. Alveolar macrophages, monocytes and neutrophils were collected and sorted from lung single cell suspensions using a BD FACS ARIA II cell sorter [76]. Cells were washed once in PBS and resuspended in RPMI 1640 supplemented with 10% FBS. Cells were distributed in 96 well plates, 100,000/well, and incubated at 37°C for 2 h. Serial dilutions of the cell suspensions were plated on LB plates before incubation at 37°C for 24 h prior to quantification of colony numbers. Visualization and quantification of uptake was also assessed using DsRed *S*. *aureus* and an AMNIS ImageStream imaging flow cytometer. Phagocytosis was assessed using DsRed *S*. *aureus*.

Reactive oxygen species were quantified using the dye 2’,7’-dichlorodihydrofluorescein diacetate (H_2_DCFDA). On day 4 of AM culture, cells were treated with 250 ng/ml of recombinant IL-12 (Peprotech) for 24 h prior to incubation with *S*. *aureus* (MOI 1) and 10 μM of H_2_DCFDA for one hour prior to flow cytometry analysis.

### RNA sequencing and analysis

RNA from monocytes was extracted using the Direct-zol RNA MicroPrep Kit (Zymo Research), following the manufacturer’s instructions. RNA-seq was performed by Novogene using the SMARTer Stranded total RNA-seq kit v3. Sequencing was performed on a NovoSeq 6000 (Illumina) with 20 million paired end 150 bp reads. Sequencing data were analyzed as follows: raw transcriptome reads were assessed for quality control (FASTQC v0.11.8) and trimmed for quality and adapter contaminant (cutadapt v 2.5). Trimmed reads were aligned to the mouse reference genome (GRCm39) using STAR (v2.6.1), followed by transcript abundance calculation and hit count extraction with StringTie (v2.0) and featureCounts (v1.6.4) respectively. Hit count normalization and differential gene expression group cross-comparisons were performed using DESeq2 (v1.26.0). Significant differentially expressed gene thresholds were set at FDR adjusted p <0.05. Pathways were analyzed using Ingenuity Pathway Analysis (Qiagen). Gene set enrichment analyses (GSEA) were performed as described previously [77], with gene lists ranked by wald statistic from differential testing and 1000 permutations performed to estimate p-values per gene set. Sequencing data has been deposited as GSE229895 in NCBI Gene Expression Omnibus.

### Statistics

Two samples were compared using an unpaired Student’s t test unless assumption of normality was violated (Shapiro Wilk test, p < 0.05), in which case Mann-Whitney tests were used. One-way analysis of variance (ANOVA) was used to assess multiple comparisons. A p-value <0.05 was considered significant. Statistical analyses were performed with the GraphPad Prism software version 9.0.1 for Windows (GraphPad, La Jolla, CA, USA). All experiments were conducted on at least two separate independent occasions.

Numerical data for all figures is included as S1 Data.

## Supporting information

S1 Fig CCR2-mediated depletion of monocytes does not adversely affect cell populations. Wildtype and CCR2-DTR mice were injected intraperitoneally with diphtheria toxin (DT). 24 h later, mice were inoculated intranasally with 10^7^ cfu of *S*. *aureus* USA300. Mice were sacrificed 24 hours later. BALF cells were stained and analyzed by flow cytometry. WT, n = 10, CCR2-DTR, n = 12. DTR = CCR2-DTR. Eosinophil difference was tested with a Mann-Whitney test. Bar graphs show mean and SEM. *p<0.05.
(TIF)

S2 Fig Purified lung monocytes from mice infected with *S*. *aureus* and bone marrow monocytes from naïve mice were analyzed by RNA-seq. Gene ontology analysis showing the top 20 canonical pathways differentially expressed between the two groups.
(TIF)

S1 Table Top 20 genes upregulated in LM vs BM across canonical pathways. (DOCX)

S2 Table Top 20 genes downregulated in infected vs naïve monocytes. (DOCX)

S1 Data All original values used to generate graphical data. (XLSX)
