# Supplementary figures and images for: Monocyte-regulated interleukin 12 production drives clearance of Staphylococcus aureus
Source: PLoS Pathog. 2024 Oct 17;20(10):e1012648. doi: 10.1371/journal.ppat.1012648 (PMC11521269; doi:10.1371/journal.ppat.1012648)

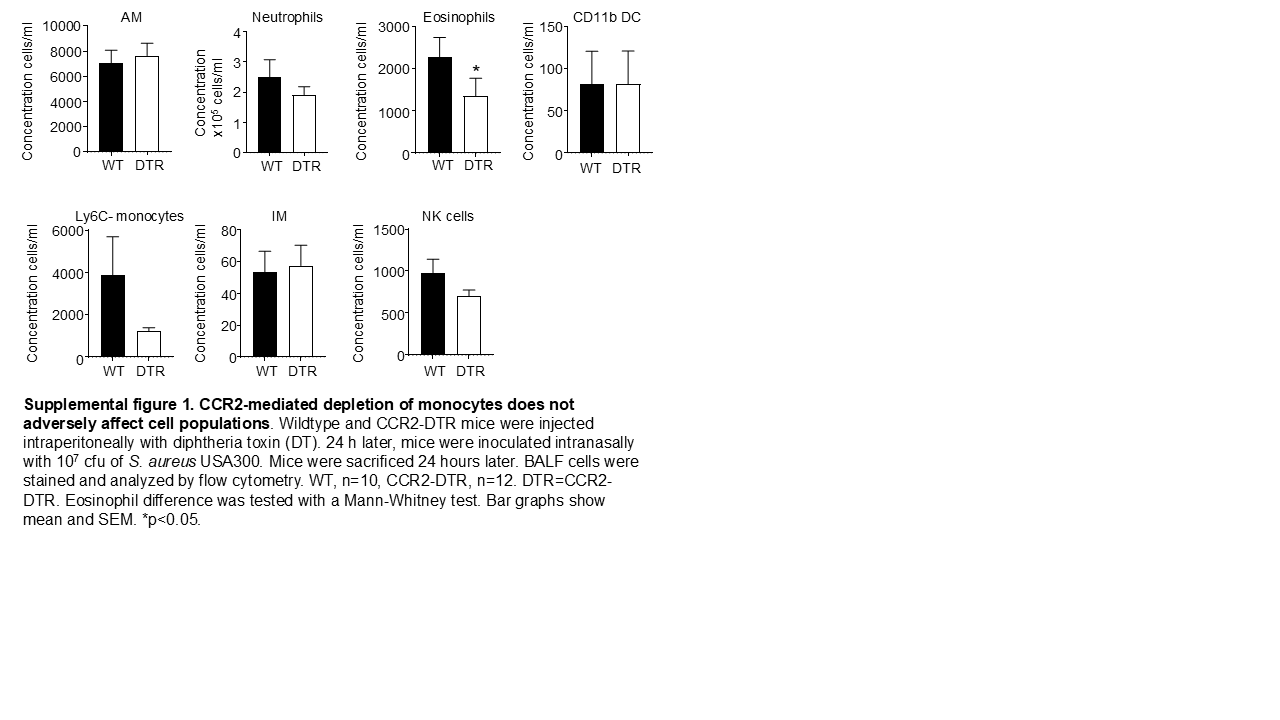

Supplement: S1 Fig — Wildtype and CCR2-DTR mice were injected intraperitoneally with diphtheria toxin (DT). 24 h later, mice were inoculated intranasally with 107 cfu of S. aureus USA300. Mice were sacrificed 24 hours later. BALF cells were stained and analyzed by flow cytometry. WT, n = 10, CCR2-DTR, n = 12. DTR = CCR2-DTR. Eosinophil difference was tested with a Mann-Whitney test. Bar graphs show mean and SEM. *p<0.05. (TIF) [file ppat.1012648.s001.tif]

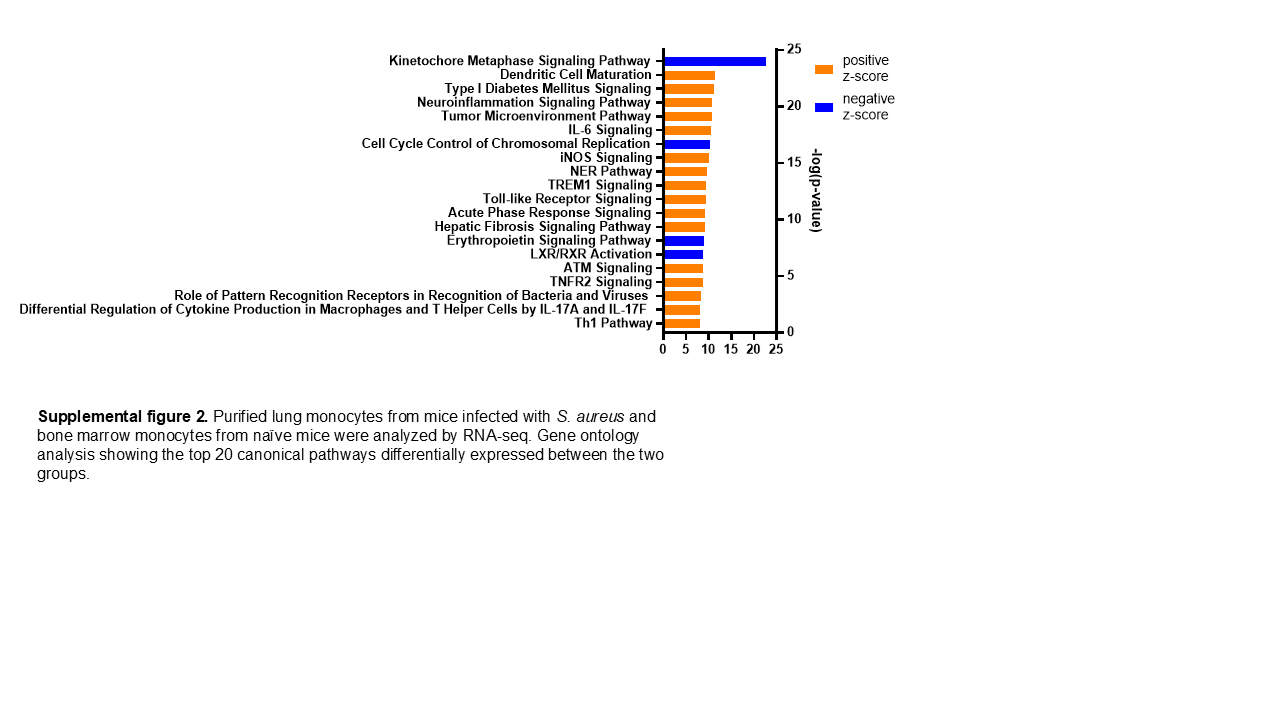

Supplement: S2 Fig — Gene ontology analysis showing the top 20 canonical pathways differentially expressed between the two groups. (TIF) [file ppat.1012648.s002.tif]
