# Supplementary material for: Monocyte-regulated interleukin 12 production drives clearance of Staphylococcus aureus
Source: PLoS Pathog. 2024 Oct 17;20(10):e1012648. doi: 10.1371/journal.ppat.1012648 (PMC11521269; doi:10.1371/journal.ppat.1012648)
Supplement: S1 Table — (DOCX) [file ppat.1012648.s003.docx]

#### Table S1: Top 20 genes upregulated in LM vs BM across canonical pathways

| Symbol | Entrez Gene Name | Expression Log Ratio | Expression p-value | Location | Type of encoded factor |
| --- | --- | --- | --- | --- | --- |
| *Cxcl3* | C-X-C motif chemokine ligand 3 | 10.966 | 5.8E-194 | Extracellular Space | cytokine |
| *Il1b* | interleukin 1 beta | 10.385 | 5.9E-240 | Extracellular Space | cytokine |
| *Socs3* | suppressor of cytokine signaling 3 | 9.254 | 1.3E-220 | Cytoplasm | phosphatase |
| *Ptgs2* | prostaglandin-endoperoxide synthase 2 | 8.288 | 1.18E-89 | Cytoplasm | enzyme |
| *Il1a* | interleukin 1 alpha | 8.013 | 2.2E-141 | Extracellular Space | cytokine |
| *Dusp1* | dual specificity phosphatase 1 | 7.881 | 7.5E-143 | Nucleus | phosphatase |
| *Slc7a11* | solute carrier family 7 member 11 | 7.594 | 7.2E-190 | Plasma Membrane | transporter |
| *Cd14* | CD14 molecule | 7.551 | 8.9E-195 | Plasma Membrane | transmembrane receptor |
| *Saa3* | serum amyloid A 3 | 7.442 | 5.1E-171 | Extracellular Space | other |
| *Il1rn* | interleukin 1 receptor antagonist | 7.418 | 1.9E-186 | Extracellular Space | cytokine |
| *Emp1* | epithelial membrane protein 1 | 6.781 | 1.84E-72 | Plasma Membrane | other |
| *Osm* | oncostatin M | 6.594 | 1.7E-125 | Extracellular Space | cytokine |
| *Il6* | interleukin 6 | 6.549 | 1.42E-22 | Extracellular Space | cytokine |
| *Tnf* | tumor necrosis factor | 6.382 | 6.9E-110 | Extracellular Space | cytokine |
| *Spp1* | secreted phosphoprotein 1 | 6.192 | 3.12E-90 | Extracellular Space | cytokine |
| *Zfp36l1* | ZFP36 ring finger protein like 1 | 6.151 | 1.54E-91 | Nucleus | transcription regulator |
| *Traf1* | TNF receptor associated factor 1 | 6.133 | 8.1E-116 | Cytoplasm | other |
| *Arg2* | arginase 2 | 6.063 | 1.74E-61 | Cytoplasm | enzyme |
| *Ets2* | ETS proto-oncogene 2, transcription factor | 6.061 | 2.37E-84 | Nucleus | transcription regulator |
| *Tnfaip3* | TNF alpha induced protein 3 | 6.026 | 4.3E-154 | Nucleus | enzyme |
