# Supplementary material for: Monocyte-regulated interleukin 12 production drives clearance of Staphylococcus aureus
Source: PLoS Pathog. 2024 Oct 17;20(10):e1012648. doi: 10.1371/journal.ppat.1012648 (PMC11521269; doi:10.1371/journal.ppat.1012648)
Supplement: S2 Table — (DOCX) [file ppat.1012648.s004.docx]

#### Table S2: Top 20 genes downregulated in infected vs naïve monocytes

| Symbol | Entrez Gene Name | Expression Log Ratio | Expression p-value | Location | Type of encoded factor |
| --- | --- | --- | --- | --- | --- |
| *Mpo* | myeloperoxidase | -8.781 | 8.8E-128 | Cytoplasm | enzyme |
| *H2bc12* | H2B clustered histone 12 | -7.294 | 1.22E-47 | Nucleus | other |
| *H4c11* | H4 clustered histone 11 | -6.421 | 1.27E-30 | Nucleus | other |
| *Bub1* | BUB1 mitotic checkpoint serinethreonine kinase | -5.591 | 1.1E-37 | Nucleus | kinase |
| *Cenpe* | centromere protein E | -5.378 | 5.51E-70 | Nucleus | other |
| *Plk1* | polo like kinase 1 | -5.216 | 6.24E-43 | Nucleus | kinase |
| *Nuf2* | NUF2 component of NDC80 kinetochore complex | -5.144 | 8.2E-40 | Nucleus | other |
| *H2bc8* | H2B clustered histone 8 | -5.027 | 1.06E-36 | Nucleus | other |
| *Knl1* | kinetochore scaffold 1 | -5.016 | 2.92E-64 | Nucleus | other |
| *Birc5* | baculoviral IAP repeat containing 5 | -4.846 | 3.14E-45 | Cytoplasm | other |
| *Ndc80* | NDC80 kinetochore complex component | -4.845 | 3.15E-36 | Nucleus | other |
| *Cdca8* | cell division cycle associated 8 | -4.793 | 4.26E-43 | Nucleus | other |
| *Cdk1* | cyclin dependent kinase 1 | -4.669 | 1.44E-33 | Nucleus | kinase |
| *Ccnb1* | cyclin B1 | -4.625 | 1.43E-36 | Cytoplasm | kinase |
| *Cenph* | centromere protein H | -4.516 | 2.77E-16 | Nucleus | other |
| *Cdc20* | cell division cycle 20 | -4.514 | 4.13E-38 | Nucleus | other |
| *Ccnb2* | cyclin B2 | -4.456 | 1.99E-44 | Cytoplasm | other |
| *Spc24* | SPC24 component of NDC80 kinetochore complex | -4.369 | 7.16E-35 | Cytoplasm | other |
| *Spc25* | SPC25 component of NDC80 kinetochore complex | -4.313 | 1.5E-28 | Cytoplasm | other |
| *Aurkb* | aurora kinase B | -4.3 | 2.09E-38 | Nucleus | kinase |
